# Pronation type Galeazzi-equivalent fracture: A rare case report (CARE-compliant)

**DOI:** 10.1097/MD.0000000000018429

**Published:** 2019-12-27

**Authors:** Seung Bum Chae, Jae Bum Kwon

**Affiliations:** Department of Orthopaedic Surgery, College of Medicine, Catholic University of Daegu, Daegu, South Korea.

**Keywords:** distal radiounlar joint, Galeazzi equivalent, Galeazzi fracture

## Abstract

**Rationale:**

Galeazzi fractures are uncommon, and are less common in children than in adults. Galeazzi-equivalent fractures, a variant of the classic Galeazzi fractures, mostly occur in children or adolescents. Galeazzi equivalent fractures may commonly accompany distal radial fractures or injury of the epiphyseal plate of the distal ulna.

**Patient concerns:**

A 13-year-old man visited our emergency department after stumbling over a rock. Manual reduction and splinting was already done in a nearby medical center. Plain radiographs revealed fractures in the distal radius and fracture of the ulnar epiphyseal plate.

**Diagnosis:**

Plain radiographs showed Galeazzi-equivalent fracture. The result of primary closed reduction was not enough and there was still displacement of fracture.

**Interventions:**

Anatomical reduction of distal radius was fixed with a plate and screws, and K-wires were inserted percutaneously for reduction and fixation of ulnar fracture.

**Outcomes:**

Complete bone union was achieved and normal range of motion is shown 2 years postoperatively. The patient is able to perform daily activities and sport activities without any signs of ulnar growth arrest.

**Lessons:**

Open reduction is required in patients with malalignment, failure to reduce the DRUJ or maintain its reduction, or older ages which are hard to expect sufficient bone remodeling.

## 1 Introduction

Galeazzi fractures are uncommon, and are less common in children than in adults. Prevalence rates in adults vary between articles, but prevalence rates have been reported to be <3% of all pediatric forearm fractures.^[1]^ The Classic Galeazzi fracture is an injury involving fracture of the radial shaft and the injury of the distal radioulnar joint (DRUJ). The mechanism of injury includes excessive rotation of the forearm and axial loading to the wrist joint, leading to fractures and injury to the DRUJ. In adults, type 1 fractures are triggered by the axial loading to the supinated wrist joint, which results in dorsal displacement of the raidus and volar dislocation of the distal ulna. Type 2 fractures are triggered by axial loading to the pronated wrist joint, resulting in volar displacement of the radius and dorsal dislocation of the distal ulna.^[2]^

Galeazzi-equivalent fractures, a variant of the classic Galeazzi fractures, mostly occur in children or adolescents. Galeazzi equivalent fractures may commonly accompany distal radial fractures or injury of the epiphyseal plate of the distal ulna. The epiphysial plates of children are biomechanically weaker than the ligaments of DRUJ.^[3]^ If external force is applied to the distal ulna, even without the rupture of the triangular fibrocartilage complex (TFCC), isolated avulsion fractures of the epiphyseal plates, and Galeazzi-equivalent fractures might occur.^[4]^

Neurovascular injuries are uncommon in Galeazzi-equivalent fractures. Protuberance of the ulnar head can be found if radial deformity is noted on inspection, and if the DRUJ injury is accompanied. Such injuries of ligaments are not easy to diagnose, and may require physical examination of the DRUJ including assessment of local tenderness or joint instability.

Similar to other forearm fractures, complications include nerve compression, entrapment of tendons, nonunion, malunion, and infection. Median neuropathy due to direct injury or traction ischemia may occur as a rare complication. Malunion of the radius, which are mostly caused by reduction failure during closed reduction of the complete radial fractures, may cause subluxation of the DRUJ. Golz et al^[5]^ have reported ulnar growth arrest in 55% of Galeazzi-equivalent fracture cases. If the patient is young enough to grow further, deformity might be triggered due to further growth of radius and the growth arrest of the ulna. Such imbalanced growth of the radius and ulna may trigger the increase of radial articular inclination in plain radiographs, and might result in dislocation of the DRUJ.

## 2 Case report

**Figure 1:**
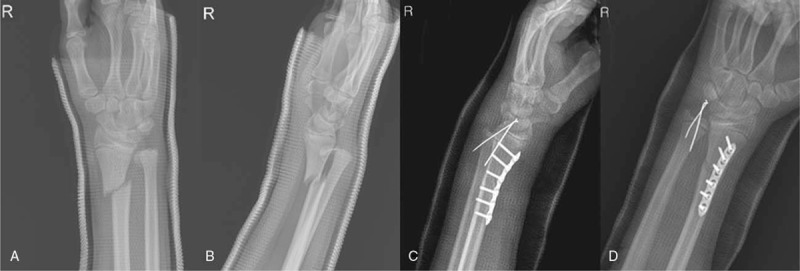
(A, B) Galeazzi equivalent fracture, pronation type. Preoperative AP and lateral views showing volar displacement of radius. (C, D) Postoperative x-rays.

**Figure 2:**
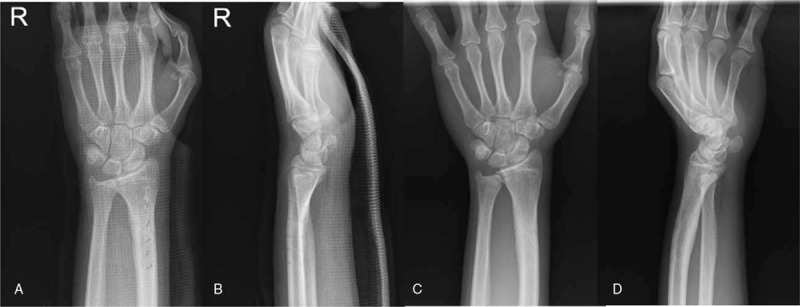
(A, B) Six months after surgery. Anatomical reduction is maintained after removal of plate and k-wires. (C, D) Two-years follow-up.

**Figure 3:**
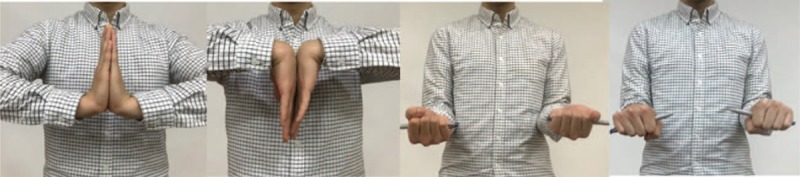
Clinical photos of wrist motions on 2-years follow-up. No limitation of motion was seen.

Patient and his family had provided informed consent for publication of the case. A 13-year-old man without any medical history visited our emergency department after stumbling over a rock. Manual reduction and splinting was already done in a nearby medical center. The patient complained of wrist pain. Plain radiographs revealed fractures in the distal radius and fracture of the ulnar epiphyseal plate (Fig. 1A, B). The physical examination showed swelling and tenderness around the right wrist. Since closed reduction was not sufficient enough, the patient was decided to undergo surgical treatment. The surgery was held at the day of visit under general anesthesia. Closed reduction was performed for reduction of the distal radius fracture, but reduction was not achieved properly. For this reason, after volar approach for anatomical reduction of the distal radius, following was the internal fixation using a 6-hole LC-DCP and 6 screws. Fracture of the distal ulnar epiphysial plate showed partial reduction after fixation of the fractured radius. K-wires were percutaneously inserted close to the fractured tip of the distal ulnar. After pushing the tip using K-wires, the fractured ulna was reduced. Two K-wires were additionally inserted for fixation. Above-elbow splint was applied for fixation (Fig. 1C, D). K-wires for ulnar fixation were removed 6 weeks postoperatively, and the metal plate for radial fixation was removed 9 months postoperatively (Fig. 2A, B). Complete bone union was achieved (Fig. 2C, D). Normal range of motion is shown 2 years postoperatively, and the patient is able to perform daily activities and sport activities also (Fig. 3). Mayo score of the patient was 95 and DASH score was 11, showing high satisfaction. Complications such as growth arrest of the ulna, joint deformity, or DRUJ instability did not occur.

## 3 Discussion

In general, closed reduction and above-elbow casting is sufficient for the treatment of Galeazzi-equivalent fractures with minimal displacement. However, failure of closed reduction due to soft tissue interposition requires open reduction.

Compression plating techniques are generally preferred for the treatment of radius fractures, rather than intramedullary nailing or cross-pinning techniques. Stable anatomical reduction of the radius is necessary for stabilization of the DRUJ.^[6]^

Failure of the reduction of ulnar epiphyseal plate injury might occur in some cases, and most of the cases were due to the interposition of the soft tissues such as the periosteum, extensor tendons, or the articular capsule.[^1^,^7^] Mitsui et al^[8]^ have reported a case of Galeazzi-equivalent fracture which volar displacement of the extensor carpi ulnaris tendon interrupted proper reduction. Close attention is needed during open reduction of the ulnar epiphyseal plate in order to avoid additional damage to the epiphyseal plate.

Additional radioulnar pinning is required when DRUJ instability persists after anatomical reduction of the fractured radius and ulna. Long arm cast immobilization should be maintained for 6 weeks postoperatively, and if possible, the radioulnar pinning should be removed 4 to 6 weeks postoperatively.

Though the acceptable range of reduction is still controversial, consideration of the patient's age is necessary since bone remodeling occurs more actively in younger patients. Volar-dorsal malalignment shows a higher tendency for active bone remodeling, while radioulnar malalignment shows a lower tendency for bone remodeling. Malrotation also show a low tendency for bone remodeling.^[9]^

To summarize, ulnar growth arrest has been reported to occur as high as in 55% of Galeazzi-equivalent fracture cases.^[5]^ Open reduction is required in patients with malalignment, failure to reduce the DRUJ or maintain its reduction, or older ages which are hard to expect sufficient bone remodeling.

Pronation type Galeazzi-equivalent fractures are extremely rare in number. In the case reported by Ashish Suthar and Ashish Kothari,^[10]^ a satisfactory reduction of the distal radius fracture was achieved by closed reduction and internal fixation by K-wires, but the reduction of the ulnar fracture failed due to interposition of the soft tissues. As a result, open reduction and internal fixation by K-wires was inevitable. Additional fixation to the DRUJ was done using K-wires. Contrary to this case, interposition of the soft tissue occurred at the distal radius. Closed reduction was enough for the fracture of the ulnar epiphyseal plate, and the surgery was performed by using the percutaneous pinning technique.

Several authors have reported preferable results with no complications, such as growth arrest, in short-term follow ups after the treatment of Galeazzi-equivalent fractures.[^8^,^10^,^11^] Open reduction was performed in most of the cases. However, studies with long-term results of the ulnar bone growth after treatment of Galeazzi-equivalent fractures lack in number, and thus, it is hard to generalize. Ulnar growth arrest occurs with a high prevalence in patients with Galeazzi-equivalent fractures.^[12]^ In a long-term follow-up trial by Cha et al,^[4]^ 8 of 10 patients showed ulnar negative variance during average follow-up periods of 18.8 months. Therefore, early confirmation of the ulnar physeal arrest by serial follow-up sessions at 6 to 12 months after trauma, and further follow-up sessions over a year in order to assess the maintenance of anatomical reduction, or the occurrence of other complications such as growth arrest or accompanying deformity, might be necessary.^[6]^ Also, additional studies for long-term results is thought to be needed.

## Author contributions

**Supervision:** Jae Bum Kwon.

**Writing – original draft:** Seung Bum Chae.

**Writing – review & editing:** Jae Bum Kwon.
